# Comparative Management Methods for Adolescents With Polycystic Ovarian Syndrome: A Systemic Review

**DOI:** 10.7759/cureus.55876

**Published:** 2024-03-09

**Authors:** Roberta L Vadan, Nanette Varela, Nikita Zhuravko, Noreena O Ogidan, Victor O Adedara, Emmanuel Keku

**Affiliations:** 1 Medicine, St. George's University School of Medicine, St. George's, GRD; 2 Public Health & Preventive Medicine, St. George's University School of Medicine, St. George's, GRD

**Keywords:** lifestyle modification, pharmaceuticals, adolescent, treatment, pcos

## Abstract

Polycystic ovarian syndrome (PCOS) is a common endocrinological disorder affecting many adolescents and women of reproductive age worldwide. A diagnosis of PCOS in adolescence relies upon investigating each medical history independently and noting commonly associated symptoms, including obesity, insulin resistance, acne, menstrual abnormalities, and hirsutism. Many researchers are aiming to discover a methodology to help manage the symptoms associated with PCOS, especially in adolescents. This review will investigate management methods possible for adolescents with PCOS. Although the most preferred way to help reduce symptoms is through lifestyle modifications such as vigorous exercise and dietary regimens low in carbohydrates, pharmaceuticals are also offering promising results to adolescents with PCOS. Metformin, oral contraceptives, gonadotropin-releasing hormone (GnRH) antagonists, and other alternatives, including finasteride, eflornithine, fibroblast growth factors (FGFs), and vitamin D, are all shown to help improve insulin sensitivity and regulate menstrual cycles and reduce hirsutism. Epilatory and surgical measurements are also available; however, they are reserved for when all other methods fail and once adulthood or an appropriate age is reached. Although there are many pharmaceuticals available, it is necessary to evaluate each adolescent with PCOS uniquely and prescribe the appropriate pharmacotherapy regarding their symptoms.

## Introduction and background

Polycystic ovary syndrome (PCOS), additionally referred to as Stein-Leventhal syndrome, is one of the most heterogeneous endocrine disorders affecting adolescent women worldwide. The prevalence of PCOS has been estimated to be around 4-21% of adolescents worldwide [1,2]. Although no particular criterion is used for diagnosis, the International Evidence-Based Guideline approved the usage of the Rotterdam Criteria (2003) for diagnosis in adult women [3]. According to this guideline, diagnosis of PCOS requires a minimum of two out of the three following features: (1) androgen excess confirmed by a blood test, (2) ovulatory dysfunction, or/and (3) 12 or more cysts on an ovary/or an ovarian volume of more than 10 mL [3]. However, adolescent diagnosis of PCOS is different in that some criteria tend to imbricate with the normal physiology of a developing female reproductive system [4]. 

PCOS is generally characterized by hormonal imbalances that occur when the ovaries produce excess androgens [1]. Moreover, it is found that PCOS has a high luteinizing hormone (LH) to follicle-stimulating hormone (FSH) ratio as well as high levels of gonadotropin-releasing hormone (GnRH) promoting the stimulation of androgen through theca cell hyperactivity. These excess hormones are detectable through the blood, resulting in menstrual irregularities, unpredictable ovulation, acne, and the formation of small follicle cysts (fluid-filled sacs with immature eggs) [1]. PCOS is often associated with comorbidities such as overweight or obesity, dyslipidemia, and hyperinsulinemia and is a risk factor for diabetes and cardiovascular diseases (CVDs) [1]. 

PCOS is a rising issue that is associated with many undesirable obstacles, and management has become a fundamental step in improving a woman's lifestyle. There are many methods of management for the treatment of PCOS in adolescents. To cope with PCOS, the most important stage is to lose at least 5% of body weight [5]. Therefore, lifestyle modifications such as having an exercise plan and diets with low levels of carbohydrates are recommended for every woman with PCOS [5,1]. On the other hand, pharmaceutical medications are used as alternatives. These include oral contraceptives, antiandrogen agents, insulin sensitizers, ovulation inducers, anti-diabetic medications, statins, and mucolytic drugs [6]. Up to this date, all the recent therapeutic options for PCOS are presently approved for indications other than PCOS. There is no current United States Food and Drug Administration (USFDA)-approved treatment, particularly for PCOS, and all mentioned medications are used off-label [6,7]. Therefore, in today's day and age, there is a need to instrument multiple beneficial options in the management of PCOS.

Although the etiology of PCOS is unidentified and specific treatment processes have not been recognized, PCOS remains one of the most common points of research and systematic inquiry in women's health. In this review, we used Preferred Reporting Items for Systematic Reviews and Meta-Analyses (PRISMA), to make an accurate assessment of the quality of the evidence being considered. Using a systemic review approach, we analyzed our search based on the setting of management options for adolescent women with PCOS from a sample size of 13,749 articles. We compared the management methods for adolescents with PCOS and postulated several commonly prescribed medications with the mechanism of each drug and lifestyle modifications that can improve the outcome of PCOS.

## Review

Methods

A comprehensive search was conducted using the following electronic databases: PubMed, Google Scholar, Web of Science, IEEE Xplore, JSTOR, and Scopus. The search was limited to English-language articles published from 1995 to 2023. The associated keywords that were used in combination to retrieve relevant articles included the following: "adolescents with PCOS", "pharmaceuticals used in PCOS", "treatment for PCOS", "PCOS and oral contraceptives," "PCOS and statin use", "PCOS and metformin".

To satisfy the inclusion criteria for this particular systemic review, we focus on (1) studies that were performed or tested on humans, (2) studies that were published in English, (3) studies that were published in peer-reviewed journals, and (4) studies that investigated PCOS diagnostic features, the pathogenesis, and treatment of PCOS. Furthermore, our systematic review excluded the following: (1) animal studies, (2) studies that were published in any language other than English, (3) non-peer-reviewed articles, and (4) studies that focused on topics besides PCOS.

**Figure 1:**
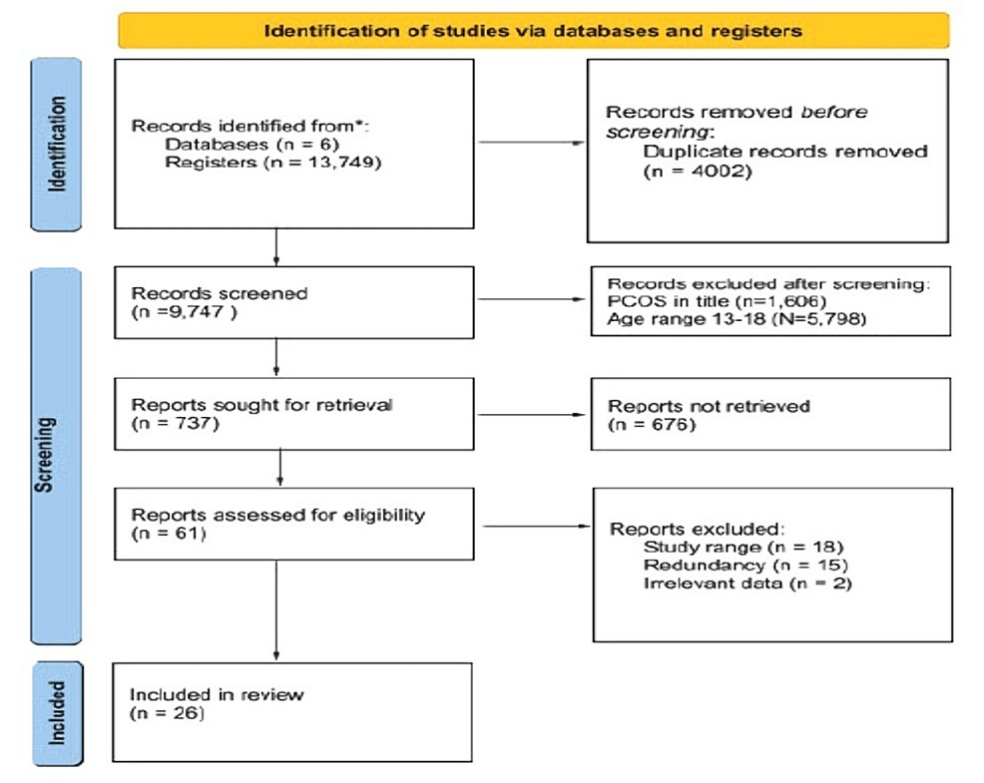
Identification of new studies via databases and registers The Preferred Reporting Items for Systematic Reviews and Meta-Analyses (PRISMA) diagram was created by the author.

Four independent reviewers screened the titles and abstracts of the articles identified through the search strategy. A total of 18 articles were removed due to being too specific in their comparison management methods, 15 provided too similar information, and two studies provided homeopathic interventions. The data from the studies and papers utilized were analyzed using a systematic approach, in that each paper had an in-depth review of qualitative data, as outlined in Figure 1.

Results

PCOS primarily affects women with high levels of androgens and dysregulation in the hypothalamic-pituitary-ovarian axis due to hormone imbalances [3]. Therefore, treatment for adolescents diagnosed with PCOS is crucial to promote menstrual regularities and reduce the risk of developing diabetes and cardiovascular disease. The right pharmaceutical treatment for each patient depends on the severity of symptoms and what the overall desired goal is: regulate menstrual cycles, reduce acne severity, and/or reduce hirsutism levels. 

There are many courses of treatment for PCOS, which include lifestyle interventions, medical interventions, surgical interventions, cognitive behavioral therapy, or a combination of treatments. Although there are many options, it is important to find the right one for the patient [8]. Adolescents with PCOS comprise a special group of people, each individual with different treatment approaches. One obstacle that physicians face when diagnosing adolescents with PCOS is that there is a high chance of overdiagnosis, due to the adult criteria overlapping with normal female reproductive system maturation [4]. Still, the most researched form of treatment is metformin. Metformin is the first-line treatment for those with type II diabetes. It is shown to regulate menstrual cycles, improve glucose levels, decrease BMI, mildly-to-moderately alleviate insulin resistance, and have relatively mild adverse effects such as gastrointestinal effects and lactic acidosis [9]. Unfortunately, its use in adolescents is still considered "off-label", but recent guidelines for the assessment and management of PCOS note that metformin, in addition to lifestyle changes, can be considered, especially in adolescents with a definitive diagnosis or clear symptoms of PCOS [9]. Witchel et al. [9] explained that, for a diagnosis of PCOS in adolescence to take place, irregular menstrual periods that occur for more than 45 days or less than 21 days need to occur for three to four years postmenarche, along with hyperandrogenism that any other disorder cannot explain. Therefore, diagnosis in each adolescent needs to consider the medical history thoroughly and uniquely for each person; PCOS can be diagnosed along with other similar features mimicking other endocrinological diseases, including hyperprolactinemia and nonclassical congenital adrenal hyperplasia (21-hydroxylase deficiency by serum 17-hydroxyprogesterone (17-OHP)) [9,1].

Oral contraceptives, specifically combined estrogen and progestin regimens, are used for menstrual cycle regulation, clinical hyperandrogenism, hirsutism, or acne. There is no set dosage regimen set, but the minimum dose given to adolescents should be 20-30 micrograms of ethinylestradiol [9]. The risks and benefits of prescribing oral contraceptives should always be considered due to the possible risk of thromboembolism formation. Many physicians resort to metformin in combination with oral contraceptives, especially in high BMI groups or those susceptible to type 2 diabetes [9].

Additionally, anti-androgen treatments can be considered, such as GnRH analogs, ketoconazole, steroids, spironolactone, or flutamide. The uses of anti-androgen therapy are mainly for the treatment of hirsutism or alopecia when other modes of treatment are contraindicated or fail [10]. For the best management of hirsutism, they should be used with oral contraceptives, or as an adjunct when oral contraceptives are not producing a clinically desired result [9]. Although this class of drug is commonly prescribed, it is important to take into consideration its adverse effects and its contraindications. One of the most significant effects is fetal undervirilization [9]. Thus, anti-androgens must carefully be used with contraception methods if the adolescent claims to be sexually active. However, even with all the risks, a recent study showed that early anti-androgen treatment is associated with an increased probability of childbirth later in life [11].

Finasteride is another anti-androgen drug that should have special consideration when considering prescribing it to adolescents. The Journal of the Endocrine Society does not recommend its use due to the data on the drug being very limited [12]. However, changes in hirsutism levels were significantly greater when combined with spironolactone, than spironolactone alone [12]. Another study by Diri et al. [12] concluded that low doses of finasteride have similar efficacy as a full dose, with the benefit of being safer and cheaper. In general, finasteride is a safe and effective treatment option for PCOS [13].

A newer alternative some physicians and scientists have turned to investigate is eflornithine. This particular drug is being reported to be a newer alternative for adolescents and women dealing with hirsutism [14]. However, it should be reserved for more serious facial hirsutism, as one of the side effects is acne [14]. 

Acne is found to be 1.6 times-fold higher in PCOS patients (43% PCOS and 21% without PCOS), and 59% in adolescents with PCOS as compared to 42% of adult PCOS patients [15]. A common therapy used to help with acne is isotretinoin referred to by the brand name, Accutane.

Isotretinoin is a vitamin A derivative and was found not only to significantly decrease acne, but also free testosterone, insulin levels, and ovarian volume [16]. Although it is associated with side effects such as increasing triglyceride and cholesterol levels, it should be considered when oral contraceptive use is not an option.

High BMI, triglycerides, and low-density lipoprotein (LDL) levels do not have a known cure. Rather, physicians primarily resort to lifestyle modifications to reduce the risk of associated cardiovascular diseases and the onset of diabetes. The most common modifications suggested by physicians are cardiovascular exercise, a diet with reduced carbohydrates, sufficient sleep, and positive mood regulation.

Gu et al. explained that insulin resistance decreased significantly with a vigorous exercise regimen compared to a moderate aerobic regimen, including a 10-minute warm-up, and 10-minute cooldown, along with 90 minutes per week of motorized treadmill running for eight consecutive weeks [17]. However, the researchers also postulated that PCOS patients are more likely to maintain a sedentary lifestyle rather than engage in vigorous workout regimens, suggesting that mood regulation and aptitude play an important role in the willingness to continue exercising. Additionally, many researchers claim that a strict diet is necessary to attain PCOS; however, there is limited evidence that one particular type of diet is better than another type [18]. However, one primarily key dietary consideration that helps reduce BMI and improve insulin resistance, as well as dietary cholesterol levels, is to reduce carbohydrate intake. Rodriguez Paris et al. discovered in their research through mice that, if carbohydrate intake is overall less than 45% of the total intake of calories, improvements in weight and cholesterol can be seen with at least a one-month consistency in this regimen [18]. With a reduction in weight and BMI, adolescents can alleviate their menstrual abnormalities and attain a balanced metabolic blood profile, including regulating blood glucose levels.

Sleep regulation in adolescents is also a highlight to help alleviate PCOS symptomatology. Sam et al. proposed that adolescents and teenagers tend to have more unorganized sleep architecture compared to any other age group. However, adolescents with diagnosed PCOS as well as obesity are shown to also have reduced rapid eye movement (REM) compared to healthy controls that are obese [19]. Therefore, ensuring that women attain at least seven to eight hours of quality sleep each night through sleep supplementation or exercise helps regulate insulin sensitivity and reduce cardiovascular risks.

Pharmaceuticals most commonly prescribed to women with PCOS rely upon reducing androgen and improving insulin sensitivity. Statins are one class of drugs that physicians use to help reduce cholesterol levels by inhibiting 3-hydroxy-3-methylglutaryl-coenzyme A (HMG-CoA) reductase, an enzyme in the formation of cholesterol. Sokalska et al. reported in their study that isolated human theca cells from the ovaries of four women with PCOS and four women without PCOS that, when treated with simvastatin or mevastatin, the proliferation of the cells reduced, with, in turn, lowered androgen production. Statins can be used from the age of eight and are recommended when all alternatives have failed or are not possible [20].

Fibroblast growth factors (FGFs) are involved in the regulation of carbohydrate and lipid metabolism and cardioprotective activity. A novel analog of FGF 21 is currently being tested, which improves blood glucose and insulin resistance, as well as cholesterol, triglycerides, and LDL [14]. Although the adverse effects are not known, FGFs have been shown to promote excessive activity of the sebaceous glands and thus should be considered when the benefits outweigh the risks for adolescents [14].

Depilatory methods, including laser hair removal, electrolysis, and thermolysis used for hirsutism treatment, can also be taken into consideration; however, an appropriate age must be reached and determined before beginning the procedure [2]. Moreover, ovarian drilling is a procedure that is routinely performed, which involves inserting a laparoscope into the abdomen and eliminating part of the ovary or ovaries affected to reduce the production of androgens [21]. However, in adolescents, it is expected to first establish medicinal and lifestyle changes. Once adulthood is reached and no alterations to symptoms can be made, it is established as a potential way to help reduce the symptoms of the disease [21]. Table 1 gives an overview of treatments that are involved in PCOS. Table 2 gives a brief review of PCOS patients.

**Table 1:** Summary of specific PCOS treatments that correspond to hormonal imbalances The table was created by the authors. PCOS: polycystic ovarian syndrome

Hormonal imbalances associated with PCOS	Specific PCOS treatments
Menstrual cycle regulation	Metformin; oral contraceptives; vitamin D
Androgen control	Oral contraceptives; eflornithine; statins; ovarian drilling
Lipidemia control (TG, LDL, cholesterol)	Statins; FGFs; lifestyle modifications
Acne control	Oral contraceptives; isotretinoin
Hirsutism control	Oral contraceptives; anti-androgens; eflornithine
Glycemic and insulin resistance control	Metformin; FGFs; vitamin D; lifestyle modifications
Increased probability of conceiving	Anti-androgens
Reduction of BMI	Metformin; lifestyle modifications

**Table 2:** A brief review of PCOS patients PCOS: polycystic ovarian syndrome; OCPs: oral contraceptive pills

Author Name, Year	Attributable findings
DiVall et al. (2019) [4]	The diagnosis of PCOS in adolescents should not use the criteria for adult diagnosis. Overdiagnosis in pediatric patients creates a burden, so different criteria should be used.
Legro et al. (2013) [5]	The Rotterdam criteria is widely used for the diagnosis of PCOS. It establishes that in order for diagnosis to take place, the following criteria should be met, which include increased androgens, polycystic ovaries, and ovulatory dysfunction.
Sadeghiet et al. (2022) [6]	The pathogenesis of PCOS relies on the increased levels of androgens. Although there is a wide variety of individualized treatments based on each female, the most common treatments include statins, metformin, DPP-4 inhibitors, GLP-1 agonists, and SGCT-2 inhibitors.
Naz et al. (2019) [7]	There is a discrepancy in the variation of the prevalence of PCOS in the population. It is determined that 11.04% of adolescent females are diagnosed with PCOS.
Hoeger et al. (2021) [8]	The diagnosis of PCOS relies on meeting many categories. Clinicians argue that earlier intervention can offer women a greater quality of life.
Witchel et al. (2019) [9]	The diagnosis of PCOS in adolescents and women can greatly vary on specific symptoms. The most common diagnostic features include hyperandrogenism and menstrual irregularities. Some females only meet some of the criteria and are therefore identified as” high risk”; however, they still require intervention with lifestyle modifications, education, and OCPs, metformin, or other medications to reduce the effects of hirsutism and acne.
Spritzer et al. (2016) [10]	Hirsutism is one of the most common symptoms associated with PCOS. The management varies; if first-line therapy with OCPs is contraindicated, management with metformin in combination with lifestyle changes is appropriate.
Elenis et al. (2021) [11]	The probability of childbirth in a woman with PCOS is 11% lower and 40% lower in women with hyperandrogenism symptoms than a woman with a diagnosis of PCOS. Comparably, women who received treatment with anti-androgens from an adolescent age had an increased probability of childbirth after spontaneous conception.
Diri et al. (2017) [12]	The administration of finasteride alone was very effective at reducing androgen levels and insulin resistance. The combination of finasteride and metformin did not have any statistically significant results. Therefore, this dual therapy was not superior to monotherapy with either finasteride or metformin alone.
Tartagni et al. (2014) [13]	Finasteride, a 5-alpha reductase inhibitor, has been shown to reduce the levels of hirsutism when a low dose is given every three days to adolescents with PCOS. This regimen has been shown to be efficacious and improve hyperandrogenism symptoms.
Rashid et al. (2021) [14]	Modified therapies for women with PCOS have extended to show promising results with DPP-4 inhibitors, GLP-1 agonists, and SGLT2 antagonists. Additional therapies with laser hair removal also improve women’s quality of life by reducing hyperandrogenism symptoms.
Ramezani et al. (2021) [15]	Among women who are diagnosed with PCOS, the most common manifestation is the development of acne. Many treatment options target the reduction and treatment of acne.
Acmaz et al. (2019) [16]	Isotretinoin or Accutane provides glorifying results for women with acne suffering from PCOS. In those with contraindications to the usage of OCPs, isotretinoin may provide more promising results in reducing cystic acne and treating long-term acne.
Gu et al. (2022) [17]	In adolescents who are diagnosed with PCOS, lifestyle modifications are the first-line therapy. Dietary, sleep, and exercise modifications all contribute to weight loss and overall reduced metabolic demand by the body.
Rodriguez Paris et al. (2020) [18]	Utilizing a mouse model, it was determined that diets that are low in protein and have medium carbohydrate and fat levels aid in decreasing the metabolic effects associated with PCOS.
Sam et al. (2019) [19]	Women diagnosed with PCOS tend to have altered sleep habits due to the increased risk of obstructive sleep apnea. Increased body weight contributes to the development of PCOS and more frequent sleep disturbances.
Sokalska et al. (2010) [20]	Statin daily medication use by women with PCOS helps to decrease the interstitial theca cells from proliferating, thereby reducing androgen levels and plasma cholesterol levels. The postulated theories about statins include reduced oxidative stress in the body and decreased de-novo cholesterol formation.
Gomel et al. (2004) [21]	Women with PCOS who have refractory anovulatory cycles with clomiphene may be offered ovarian drilling. With this procedure, the stroma of the ovaries is destroyed to reduce the effects of androgen in the body and, therefore, induce ovulation.
Johnson (2014) [22]	Insulin resistance is just one of the main consequences associated with PCOS. The diabetic drug Metformin can be used to induce ovulation in non-obese women and can be used as an alternative to OCPs to treat hirsutism and acne and prevent type 2 diabetes and cardiovascular disease. In women with PCOS interested in in-vitro fertilization, administering metformin is also beneficial to prevent ovarian hyperstimulation syndrome.
Shah et al. (2018) [23]	For women with PCOS who are not ready to conceive, OCPs are the first-line therapy to help with the symptoms of acne hirsutism and regulate menstrual cycles. Although the long-term effects are highly desired, it is important to note that consequences include increased thrombosis risk, reduced bone mineral density in adolescents, and visual disturbances.
Trummer et al. (2019) [24]	Women who were given vitamin D supplements significantly decreased plasma glucose during the oral glucose tolerance test (OGTT) but otherwise had no impact on the endocrinological somatic effects.
Kim et al. (2022) [25]	Amongst women with PCOS, those who were able to incorporate daily lifestyle modifications with exercise and dietary changes effectively had the greatest improved menstrual cycles and regulated insulin than monotherapy with either just exercise or diet changes.
Zhuang et al. (2010) [26]	The increased prevalence of depression seen amongst women with PCOS is contributing to a reduced quality of life. Usage of antidepressants is shown to be effective for women with PCOS as their mood elevation provides greater motivation for them to seek treatment and change their lifestyle.

Other novel methods of PCOS treatment are also being investigated. One study suggests that vitamin D deficiency plays a role in insulin resistance, as well as in improving menstrual irregularity [14]. Moreover, Trummer et al. [24] concluded that vitamin D supplementation does not have a significant effect on metabolic and endocrine parameters, but does reduce plasma glucose during oral glucose tolerance tests. Therefore, newer alternatives are also being put into practice to discover their efficacy on more individuals and adolescents with PCOS.

Discussion 

PCOS is a multi-faceted metabolic syndrome that impacts many women today. The diverse implications it can have on patients encourage extensive research and varied treatment options. This systematic review explores several treatment approaches, their effectiveness, and considerations for healthcare providers as they seek to assist adolescents in managing PCOS symptoms and improving their quality of life.

Metformin, a well-known diabetic drug, is often considered to treat certain symptoms of PCOS, such as insulin resistance and infertility. A study was conducted by Cochrane Review where metformin was used as an ovulation induction agent in seven randomized control trials. It was reported that the group of women who took metformin had an increased pregnancy rate compared to those given a placebo. However, the study proved to be underpowered and no significant benefit was concluded [22]. In the future, more studies will need to be conducted to conclude the viability of metformin use regarding fertility in PCOS patients.

Oral contraceptives can be used to treat symptoms of PCOS, such as menstrual cycle irregularity, acne, and hirsutism. However, a benefit-risk analysis must be conducted before clinicians use oral contraceptive pills (OCPs) to treat adolescents due to possible adverse effects. A study reported by Shah et al. [23] on behalf of the National PCOS Working Group renders a consensus statement regarding the use of OCPs in PCOS patients in India. A team of endocrinologists, dermatologists, gynecologists, public health clinicians, and researchers investigated to determine its safety and efficacy to guide Indian practitioners. From their research, they concluded that low doses of combined OPCs containing neutral or anti-androgenic progestins should be heavily considered when treating PCOS symptoms, as they are the most favored among OCPs. However, this study recommends that the use of OCPs be limited to patients less than 14 years of age or who are within two years of menarche. If the benefits do outweigh the risks for this sub-cohort, the use of drospirenone, norgestimate, and norethindrone is suggested [23]. However, in every PCOS case, individualized treatment is needed, and risk factors and unique experiences should be considered.

The treatment of hirsutism is heavily dependent on the amount of psychosocial distress experienced by the patient. If combined oral contraceptives are contraindicated, metformin, along with lifestyle changes, may decrease androgen secretion and resulting hirsutism. If hirsutism is mild, non-pharmacological methods are often encouraged. However, antiandrogens and a safe contraceptive method can be used in severe cases [20]. There are other hair removal treatments such as laser hair treatment, electrolysis, shaving, waxing, and depilatories. Nonetheless, at this point, only electrolysis is FDA-approved for permanent hair removal.

Obesity is often associated with PCOS, and it has been documented that the risk of patients with PCOS having an elevated BMI is four times higher than healthy controls [24]. As increased body weight correlates with insulin resistance and increased androgens, it is a worthwhile endeavor to assist PCOS patients in achieving healthy body weight [25].

It has been found that a moderate aerobic regimen consistently adhered to can lower insulin resistance [17]. However, significant lifestyle changes in diet and exercise may be difficult to maintain if patients are depressed. It has been reported that depression in patients with PCOS is prevalent, even up to four times more than non-PCOS-affected controls. Remarkably, this statistic correlates with the prevalence of obesity in patients with PCOS. As PCOS can negatively impact self-image and cause subfertility, one can postulate that the use of SSRIs or SNRIs may be a valid option to improve mood disorders among affected adolescents [5]. Currently, there is no evidence suggesting that antidepressants are effective and safe in treating depression in patients with PCOS, as highlighted in a study by Zhuang et al. [26]. However, this may be an area of interest for clinicians to explore in the future.

## Conclusions

PCOS lacks standardized therapy according to existing medical guidelines. Given the individual variations in symptoms, tailored attention and unique therapeutic approaches are essential. Lifestyle interventions, encompassing balanced nutrition and vigorous cardiovascular exercise prove to be beneficial by reducing BMI, enhancing glucose levels, and regulating menstrual cycles. While lifestyle adjustments are pivotal, pharmaceutical management with metformin, oral contraceptives, GnRH antagonists, and emerging alternatives such as eflornithine, FGFs, and vitamin D supplementation, also play a significant role. Combining therapies, and considering symptom severity, becomes imperative in medication decisions. Beyond medicinal agents, hyperandrogenism symptoms can be addressed through epilation procedures, with surgical measures generally reserved for full reproductive system maturation. This systematic review underscores the need for individualized pharmaceutical strategies in PCOS treatment, emphasizing the potential of combined treatment regimens and the importance of further research in areas such as combining mood-altering medications with lifestyle modifications and pharmaceuticals, as well as exploring enhanced genetic features for early preventative strategies.

Although several clinicians have different opinions regarding the management of PCOS, it is important to be able to tailor unique treatment approaches toward each patient. One of the most important achievements in women with PCOS is the possibility to still be able to conceive and be given the possibility to start a family.
